# Olive Oil: Nutritional Applications, Beneficial Health Aspects and its Prospective Application in Poultry Production

**DOI:** 10.3389/fphar.2021.723040

**Published:** 2021-08-25

**Authors:** Rana M. Bilal, Chunjie Liu, Haohan Zhao, Yanzhou Wang, Mayada R. Farag, Mahmoud Alagawany, Faiz-ul Hassan, Shaaban S. Elnesr, Hamada A. M. Elwan, Huajiao Qiu, Qian Lin

**Affiliations:** ^1^ Institute of Bast Fiber Crops, Chinese Academy of Agricultural Sciences, Changsha, China; ^2^ University College of Veterinary and Animal Sciences, The Islamia University of Bahawalpur, Bahawalpur, Pakistan; ^3^ Hunan Deren Husbandry Technology Co., Ltd., Changde, China; ^4^ Forensic Medicine and Toxicology Department, Veterinary Medicine Faculty, Zagazig University, Zagazig, Egypt; ^5^ Poultry Department, Faculty of Agriculture, Zagazig University, Zagazig, Egypt; ^6^ Institute of Animal and Dairy Sciences, Faculty of Animal Husbandry, University of Agriculture, Faisalabad, Pakistan; ^7^ Poultry Production Department, Faculty of Agriculture, Fayoum University, Fayoum, Egypt; ^8^ Animal and Poultry Production Department, Faculty of Agriculture, Minia University, El-Minya, Egypt

**Keywords:** Health, olive oil, poultry, nutrition, feed

## Abstract

Plant polyphenols have promoting health features, including anti-mutagenic, anti-inflammatory, anti-thrombotic, anti-atherogenic, and anti-allergic effects. These polyphenols improve the immune system by affecting the white blood cell proliferation, as well as by the synthesis of cytokines and other factors, which contribute to immunological resistance. Olive trees are one of the most famous trees in the world. Whereas, olive olive oil and derivatives represent a large group of feeding resource for farm animals. In recent years, remarkable studies have been carried out to show the possible use of olive oil and derivatives for improvement of both animal performance and product quality. In vivo application of olive oil and its derived products has shown to maintain oxidative balance owing to its polyphenolic content. Consumption of extra virgin olive oil reduces the inflammation, limits the risk of liver damage, and prevents the progression of steatohepatitis through its potent antioxidant activities. Also, the monounsaturated fatty acids content of olive oil (particularly oleic acid), might have positive impacts on lipid peroxidation and hepatic protection. Therefore, this review article aims to highlight the nutritional applications and beneficial health aspects of olive oil and its effect on poultry production.

## Introduction

Plant-derived supplements are usually used to improve the public health and growth performance of animals (Elwan H. A. M. et al., 2019; Alagawany et al., 2019). The active molecules of plant seed oils can activate the immunity and enhance the secretion of digestive enzymes (Reda et al., 2020; Alagawany et al., 2021. (Nutrients and phytochemicals, especially polyphenols and fatty acids have shown to improve the immune system, rendering the development of dietary approaches for non-pharmacological prevention and management of the diseases (Alagawany et al., 2020a; El-Tarabily et al., 2021). One of such non-pharmacological substances is olive (*Olea europaea* L.) fruit and its by-products, including olive oil, which is isolated by the physical methods or by solvent extraction or reorganization processes. The European Union (EU) regulations (EEC Regulation 1513/2001; EU Regulation No. 29/2012 and EU Regulation 1348/2013) classify and define olive oil into different types such as: Virgin olive oil (VOO), Extra Virgin olive oil (EVOO), refined olive oil, olive oil, olive-pomace oils and lampante olive oil. Particularly, EVOO containing glycerol or saponifiable fraction represents about 90–99% of the oil. Fatty acids represent the major portion of the saponifiable compounds, mainly including monounsaturated fatty acids (MUFAs), where oleic acid make up to 80% of the total oil. Polyunsaturated fatty acids (PUFAs) constitute 3–22% of the olive oil, where saturated fatty acids (SFAs) and linoleic acid from 8 to 26% of it (Quintero-Flórez et al., 2015). Moreover, olive oil also contains minor phytochemical compounds that have many biological functions and represent 1–2% (La Lastra et al., 2001).

**FIGURE 1:**
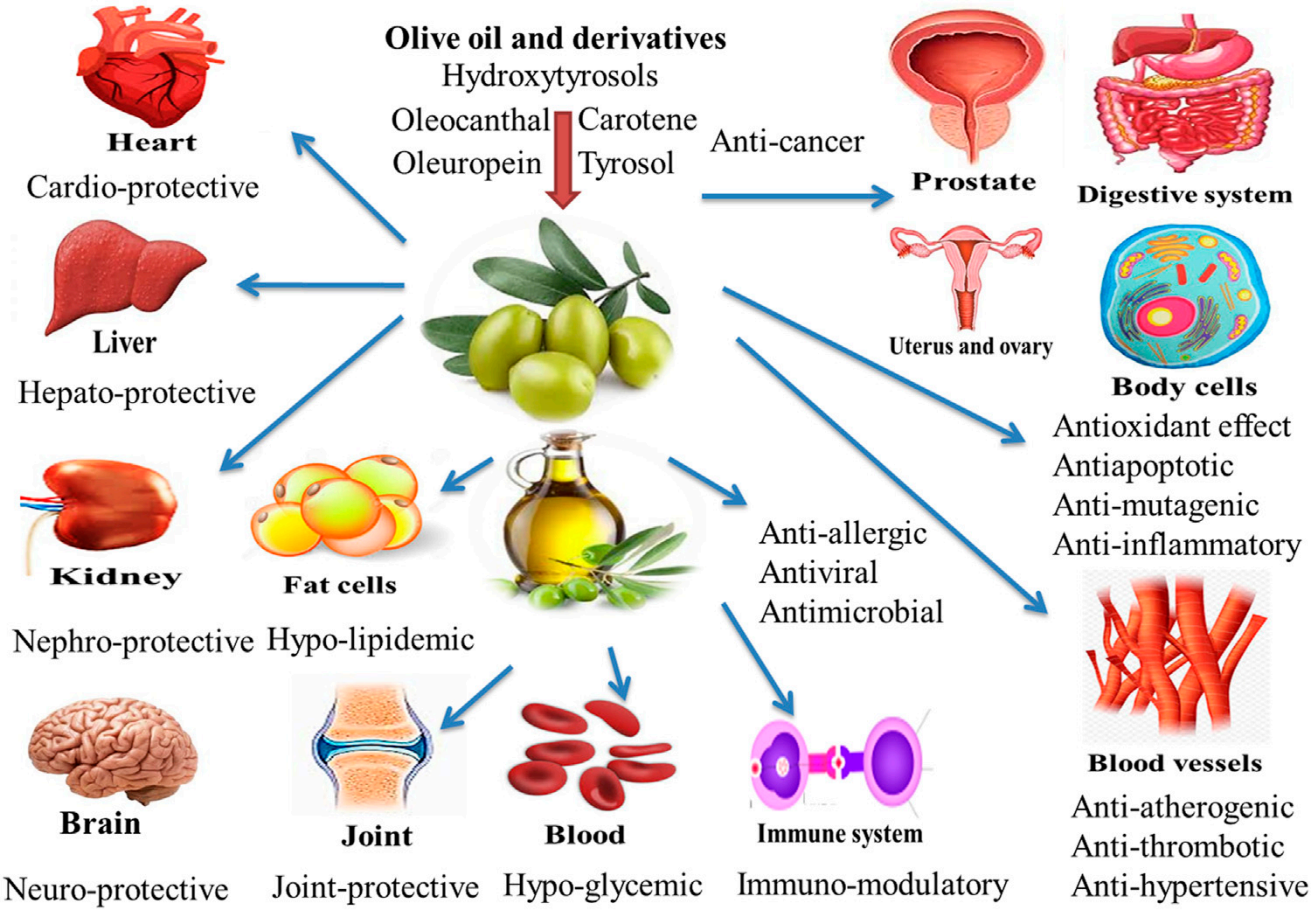
Health benefits and nutritional applications of olive oil.

The positive effects of EVOO are attributed to its higher MUFA contents, especially oleic acid, which has shown several favorable properties (Bermudez et al., 2011). MUFAs have the ability to modulate the immune response and can be useful in treating certain autoimmune diseases and in general regulation of immunity (Miles and Calder, 2015). Polyphenols of olive oil may be associated with some properties, including hypoglycemics, anti-atherogenic, antitumor, anti-inflammatory, immunomodulatory, and antiviral properties which are partly attributed to the antioxidant effect of these products (Rigacci and Stefani, 2016). Also, hydroxytyrosol (HT) ((3,4-Dihydroxyphenyl)ethanol) is a polyphenol found in extra virgin olive oil (EVOO) and red wine. It has a strong antioxidant effect due to hydrogen donation, and it can enhance radical stability. Humans, as well as cellular and animal models, have been researched for the positive benefits of HT, most notably in connection to EVOO intake. Aside from its antioxidant potential, this polyphenol has been linked to a slew of other benefits. The purpose of this study was to evaluate the major characteristics of HT for human health, with a focus on those linked to the potential prevention and/or treatment of noncommunicable illnesses (Echeverría et al., 2017). Besides, unsaturated fatty acids can perform critical biological activities such as anti-persistent role and positive impacts on endothelial function and regulation of specific parameters for inflammatory diseases (Cárdeno et al., 2014). A variety of wastes and byproducts are produced during the olive oil processing process. The main ones with significant nutritional and technological interests are olive pomace, olive mill waste waters, olive leaves, and olive stone and seed (Nasopoulou and Zabetakis 2013; Nunes et al., 2016; Nasopoulou et al., 2018). In this review, we reviewed existing information of olive oil and derivatives positive health uses and prospective effects on poultry production in this review (Figure 1).

## Methodology and Criteria Used

The current review was based on literature and patents already available on various scientific databases highlighting the nutritional applications and beneficial health aspects of olive oil and its effect on poultry production. The databases included under study were PubMed, Medline, PubMed Central, Science Direct and few other scientific databases. The information obtained through these diverse databases is compiled, critically interpreted and presented in the current study. The following inclusion criteria were used: 1) articles from any year, referring to any country; 2) articles that contained the clear information; and 3) articles in English.

### Anti-Inflammatory Activity of Olive Oil

Inflammation plays a driving role in the pathogenesis and prevalence of the joint degenerative diseases. Hence, it is imperative to control inflammation through proper pharmacological measures. The regular consumption of olive oil may alter inflammatory markers and cytokines associated with coronary artery disease (Patrick and Uzick, 2001). These beneficial and health-promoting effects of olive oil are attributed to its polyphenolic contents which have potent anti-mutagenic, anti-inflammatory, anti-thrombotic, anti-atherogenic and anti-allergic effects. Plant polyphenols have shown to decrease the morbidity or/and slow down the development of neurodegenerative and cardiovascular diseases. Approximately, 36 phenolic constituents are found in EVOO, including mainly oleocanthal, carotenes, hydroxytyrosols, oleuropein and tyrosol. These beneficial compounds, when get entry into the brain, exhibit neuroprotective actions by their potent anti-inflammatory, antioxidant and antiapoptotic properties (de Souza et al., 2017; Shi et al., 2017; Farooqui and Farooqui, 2018). A concentration of 284–711 mg/kg oleocanthal is present in EVOO. Oleocanthal plays an exclusive role in perpetual anti-inflammatory characteristics. Oleocanthal has gained attention of scientific community due to its well-known pharmacological properties. It has been found to inhibit the spread of neuro-degenerative and joint-degenerative diseases through various mechanisms. Oleocanthal in conjunction with other phenolics exhibits a neuro-therapeutic potential, which ultimately reduces neurodegenerative diseases in populations that consume EVOO on a regular basis. Hence, a continuous intake of EVOO is advised by researchers to promote human health (Parkinson and Keast, 2014).

Ibuprofen is well recognized to show a positive impact on the markers of neurodegenerative disease (Parkinson and Keast, 2014). Cyclooxygenase (COX) is a kind of oxidoreductase enzyme involved in the regulation of platelet and kidney. However, the long term use of low doses of ibuprofen and other COX cause severe side effects leading to strong inflammatory effects. *In-vivo* and *in-vitro* reports suggest that the phenolics from EVOO positively influence the inflammation, antioxidant status, and antimicrobial activity (Cicerale et al., 2012). Extra virgin olive oil contains compounds which can induce a localized irritation in the oropharyngeal region because of the perceptual similarities, hence produce an anti-inflammatory action similar to Ibuprofen (Beauchamp et al., 2005). The use of naturally occurring non-steroidal anti-inflammatory drugs (NSAIDs), like oleocanthal, may alleviate inflammation and contribute towards a substantial reduction in the development of chronic inflammatory diseases (Parkinson and Keast 2014). Similarly, oleocanthal has been considered as natural NSAIDs for an effective cure for the degenerative joint disease (Scher et al., 2007). Indeed, oleocanthal displays an anti-inflammatory action in the body equivalent to ibuprofen, as it works on exactly the same pathways as a non-steroidal anti-inflammatory drug. Hence, EVOO is a potential NSAID replacer of ibuprofen (Beauchamp et al., 2005).

Frying results in the production of free radicals in conventional cooking oils, which cause inflammation in the body after consumption. Generally, the Mediterranean people use 25–30 ml of vegetable oil in cooked foods and salad dressings (Corona et al., 2009). An excessive use of conventional oil in food products can cause substantial increase in body weight (BW), and a plethora of obesity related diseases. However, olive oil has been found beneficial in counteracting the obesity-related diseases (Scoditti et al., 2019). The higher price of EVOO than conventional cooking oils limits its frequent use. However, its therapeutic benefits make it a cost-effective alternative in the long run. EVOO is much resistance to high temperatures and contains essential antioxidant compounds which scavenge free radicals, hence perform potent anti-inflammatory actions (Dick, 2018). *In vivo* study by Molnar et al. (2021) stated that olive oil at a dose of 0.3 g/animal/day had the cell membrane protective and anti-inflammatory effects. Due to its effective anti-inflammatory action, regular intake of olive oil has shown provide relief rheumatoid arthritis, which is an autoimmune disease characterized by inflammation and pain of joints (Wahle, 2004). Moreover, the combined intake of fish and olive oils effectively controlled rheumatoid arthritis as compared to the intake of dietary fish oil alone (Berbert et al., 2005).

### Chemopreveintive Effects of Olive Oil

Olive oil has been reported to provide protection against leukemia in children and many types of cancer, such as the colon cancer and esophageal squamous cell cancer (Mosby et al., 2012). The dietary olive oil has shown to reduce the number of cancerous lesions and number of tumors (Grosso et al., 2013). It has been observed that the fatty acids present in olive oil can reduce the production of prostaglandins which in turn, can potentially inhibit the tumor development and production (de Souza et al., 2017). MUFAs of olive oil have the ability of positively altering the fatty acid profile in the animal body (Stark and Madar, 2002; Nakbi et al., 2010). Chemo-preventive property of olive oil has been associated with its phenolic contents, including hydroxytyrosol (3, 4-dihydroxyphenylethanol), (p-hydroxyphenylethanol) tyrosol and phenolic alcohols, as well as their secondary derivatives p-HPEA-EDA (oleocanthal), oleuropein, p-HPEA-EA (ligstroside aglycon), 3,4-DHPEA-EA (oleuropein aglycon) and 3,4-DHPEA-EDA (Bendini et al., 2007). Moreover, polyphenols have shown to slow down the development and progression of cancer. These polyphenols can modify the immune system through proliferation of white blood cells as well as by the synthesis of cytokines and other factors, which contribute to the immunological resistance (Ding et al., 2018). Various *in vivo* and *in vitro* reports have suggested that the olive oil might reverse or inhibit cancer progression owing to its phenolic and polyphenolic contents which scavenge free radicals or other reactive oxygen species (Wahle et al., 2010). Dietary EVOO has shown to decrease the incidence of different types of cancer such as breast, prostate and digestive system cancers (Psaltopoulou et al., 2011).

Oleocanthal, a kind of natural phenolic compounds present in EVOO, is a robust anti-inflammatory agent (Beauchamp et al., 2005). It has become a center of interest in cancer prevention programs, because it can be used as a potent natural *cyclooxygenase* (*COX*) inhibitor. It has the capability of breaking the inflammatory cascade by reducing the secretion of *COX* inflammatory enzymes (Zarghi et al., 2011).

*Hydroxytyrosol* (3,4-dihydroxyphenylethanol, HTyr) is another powerful polyphenol obtained from olives and its derived products that has been reported to exert anticancer activities (Vilaplana-Pérez et al., 2014). In mice, no mortality or morbidity was found with an aqueous extract of olive-pulp containing pure HTyr (about 1,400 mg/kg mg/day for 90 days) (Christian et al., 2004). The *HTyr* exerted anti-proliferative impacts on human *colon carcinoma cell* lines. Moreover, it significantly promoted the regulation of glomerular filtration rate in human with colorectal adenocarcinoma (Terzuoli et al., 2016). Furthermore, *HTyr also* acts as an effective cytotoxic factor against cancer cell lines in the breast. It arrests the cell cycle in the G0/G1 stage by lowering the concentration of cyclin D1 (Han et al., 2009).

Oleuropein inhibited the cell growth and induced apoptosis in different cancer cell lines (Emma et al., 2021). Oleuropein (125  mg/kg of diet), another potent polyphenol, has anticancer property. It has shown anticancer activties in human cancer cell lines (Sepporta et al., 2014). Oleuropein possesses potent anti-breast cancer properties, as demonstrated against the *mammary tumor MCF-7 cell line* (Hassan et al., 2013; Sepporta et al., 2014). The anti-breast cancer property of oleuropein is attributed to cytochrome P450 enzyme, which is a potent aromatase inhibitor and is a significant pharmacological target in the breast cancer therapy (Neves et al., 2007). Furthermore, it has shown to enhance (>1000-fold) the sensitivity of trastuzumab-conditioned SKBR3/Tzb100 breast cancer cells (Rigacci and Stefani, 2016).

### Antimicrobial Activity of Olive Oil

An *antimicrobial* agent is a substance that kills or inhibits the spread of microorganisms. Polyphenols are bioactive molecules that have been well documented for their antimicrobial and antioxidant activities (Zbakh and Abbassi, 2012). *In vitro* studies have shown that olive oil contains substantial phenolic contents, with strong antimicrobial and antioxidant activities capable to reduce the growth and propagation of several bacteria. The HTyr content of olive oil has the capability of reducing the growth of a variety of harmful microorganisms. The beneficial properties of olive include some antiatherogenic, hypocholesterolemic, antitumor, antihypertensive, cardioprotective, anti-inflammatory, antiviral, antimicrobial, antioxidant and hypoglycemic properties (Cayan and Erener, 2015). A remarkable antimicrobial response with the intake of olive extract has been attributed to its polyphenolic contents (Ritchason, 2000). Oral doses of pulverized olive leaves have been used to treat the malarial infections (Benavente-Garcõa, 2000). Polyphenols have shown to negatively affect the growth and propagation of *Bacillus cereus* and *Klebsiella pneumoniae, Salmonella typhi* and *Escherichia coli* (Appendini and Hotchkiss, 2002). Moreover, the growth and propagation of both Gram negative Pseudomonas syringae and Gram positive Corynebacterium michiganense have also been successfully inhibited by the treatment with co-products derived from the processing of olive oil (Capasso et al., 1995). *In vivo* application of olive oil and its derived products indicated a positive oxidative balance due to the presence of polyphenols (Soni et al., 2006). *Dietary olive oil showed a possible antimicrobial activity against intestinal and respiratory infections (* Sudjan et al., 2009). Campylobacter induces food related human campylobacteriosis (European Food Safety Authority, 2015).

### Hepatoprotective Activity of Olive Oil

The liver is a multifunctional organ which regulates the internal chemical environment of the body (Thirumalai et al., 2011). The liver controls the absorption and metabolism of medications and other xenobiotics in the body by purifying and removing them, hence, protects the body against external contaminants (Saleem et al., 2010). Ortiz et al. (2020) indicate that the hydroxytyrosol prevents the development of liver steatosis and the associated mitochondrial dysfunction induced by high-fat diet. The liver is often affected by free radicals, which induce the onset of liver cancer, cirrhosis, hepatitis and other diseases (Ilavenil et al., 2015), which lead to morbidity and mortality. These issues highlight the need to explore the potential of plant-derived products as hepatoprotective and therapeutic agents (Saeed et al., 2021). Historically, olive oil has been a primary ingredient in the Mediterranean diet. The Mediterranean diet has been suggested to prevent the metabolic syndrome associated with the liver (Martínez-González and Sánchez-Villegas, 2004). Antioxidant properties of olive oil play a remarkable role in reducing the malignant neoplasms (Rodriguez-Rodriguez et al., 2006). Moreover, anti-inflammatory and antioxidant properties of olive oil are useful in protecting the humans from various ailments (Fang et al., 2008). Another favorable influence of the Mediterranean diet is its rich energy contents mainly derived from MUFAs portion of olive oil. The palmitate and oleate are main fatty acid esters found in the normal liver. The proportion of linoleate and linolenic acids was reduced in patients that suffer from alcoholic fatty liver. Moreover, concentration of oleate is higher in the normal liver as compared to fatty liver and after liver biopsies (Aghdassi et al., 2007). Moreover, dietary MUFAs (from olive oil) have shown to control the hepatic steatosis mainly through activation of PPARα and PPARγ by reducing insulin resistance while enhancing lipid oxidation (Soriguer et al., 2006). EVOO contains oleic acid and polyphenols, which have been reported to exert protective impacts on the liver in several experimental models, particularly in animal studies and cell cultures (Pirozzi et al., 2016) These components of olive oil have also shown to prevent various hepatic disorders, like hepatic fibrinogenesis, hepatocyte ballooning, and liver steatosis, hence aid in prevention of hepatic tissue damage induced by CCl4 (0.6  ml/kg, intraperitoneally (i.p.)) twice a week for 6 weeks (Han et al., 2016). The MUFAs in EVOO play a pivotal role in the treatment and prevention of liver steatosis, induced by CCl4 (0.6 ml/kg, intraperitoneally (i.p.)) twice a week for 6 weeks, both alone or in combination with other components such as n-3 PUFAs particularly ‏docosahexaenoic acid (DHA, C22:6 n-3) and eicosapentaenoic acid (EPA, C20: n-3) (Valenzuela et al., 2016). The consumption of EVOO reduces the inflammation, inhibits the risk of liver damage, and prevents the progression of steatohepatitis through the antioxidant action of its polyphenolic contents (Rincón-Cervera et al., 2016). Moreover, it has also shown to remarkably control the CCl_4_-induced liver cirrhosis in a rat model. Moreover, Wang et al. (2014) demonstrated that EVOO consumption reduced the derangements in hepatic tissue, as well as decreased the formation of fibrous tissue in rats intoxicated with (0.1 ml/100 g body weight, 1:1 mixed with soybean oil) CCl_4_. The mechanisms responsible for decreasing hepatic fibrosis by using EVOO include reduction in lipid peroxidation and expression of α-smooth muscle actin (α-SMA), a protein that participates in the structure of cell (Wang et al., 2014). Another anticancer effect of polyphenolic contents of EVOO was also reported by Vilaplana-Pérez et al. (2014). Moreover, the risk of hepatocellular carcinoma was limited through inhibition of the enzyme xanthine oxidase by HTyr accompanied by a decline in superoxide anion production, which protected against DNA damage (Zhao et al., 2014). Another study reported that about 10–80 µM of HTyr precursor oleuropein found in EVOO when added to human hepatoma cell lines exhibited a dose-dependent increase in the cellular apoptosis, as well as inhibition of colony formation and cell growth, which resulted in the PI3K/AKT pathway inactivation (Yan et al., 2015). Sánchez-Calvo et al. (2021) reported a clearly imply a link between the production of NO_2_-OA from EVOO and the observed improvement in mitochondrial function in NAFLD. NO_2_-FA formation may be responsible for the health advantages linked with EVOO intake.

Ischemic/Reperfusion (I/R) is a condition in which an organ is temporarily or permanently deprived of blood flow for a specific duration of time. In the ischemic condition of the liver, there is less supply of oxygen to hepatic tissues. Moreover, there is a transformation of hepatocellular metabolism to anaerobic pathways that persuades a pro-inflammatory condition, which makes the tissue susceptible to reperfusion (Jaeschke, 2003). The constituents of EVOO have been reported to reduce the hepatic I/R injury (Pan et al., 2013). Moreover, combined treatment of olive oil with camel milk in mice exhibited hepatoprotective action against single dose (500 mg/kg) of acetaminophen-induced hepatotoxicity owing to the pronounced antioxidant action (Ibrahim et al., 2017).

Regarding, liver steatosis progresses into non-alcoholic steatohepatitis (NASH) under persistent oxidative stress conditions, (Videla et al., 2004; Rolo et al., 2012) a last-stage disease of the liver, the AR EVOO supplementation provides an appropriate therapeutically strategy to prevent or resolve the development of liver steatosis. An iron-rich diet causes oxidative stress in the liver, with increased lipid peroxidation and protein oxidation, responses associated with mitochondrial dysfunction and membrane unsaturation, the latter effect triggering a drastic increase in the SREBP1c/PPAR- ratio with the development of hepatic steatosis, thus representing a type of nonalcoholic fatty liver disease (NAFDL). In this situation, AR-EVOO supplementation corrected IRD-induced alterations, resulting in an efficient anti-steatotic natural product, the protective benefits of which may be attributed to the molecular pathways established by its primary components, namely, HT, OA, tocopherols, and PUFAs (Barrera et al., 2018).

The oxidative stress is defined as an imbalance of metabolic and radical constituents called reactive (chlorine, oxygen or nitrogen) species at the cellular level (Elwan H. et al., 2019; Elnesr et al., 2019). Olive oil has been shown its excellent potential to effectively mitigate liver oxidative stress through regulation of various pathways (Kyle et al., 1987). The rich MUFAs content of olive oil (35% ethanol solution of 3 g/kg body weight) positively influenced the lipid profiles and peroxidation of hepatic mitochondria in rabbits (Kasdallah-Grissa et al., 2008). No doubt that EVOO has remarkable health boosting properties, which are mainly associated with its abundant MUFAs and polyphenolic contents. Studies have shown that olive oil enhances circulating lipoproteins which are less sensitive to peroxidation hence decreases risk of onset of diseases in the human body (Kasdallah-Grissa et al., 2008; Necib et al., 2013). Similarly, Berrougui et al. (2015) reported that cholesterol efflux, a way of transferring intracellular cholesterol and improvement in the high-density lipoprotein-cholesterol is observed in individuals consuming the olive oil. Ortiz et al. (2020) indicated that the combination of docosahexaenoic acid and hydroxytyrosol prevents the development of liver steatosis and the associated mitochondrial dysfunction caused by a high-fat diet, highlighting the importance of this protocol as a therapeutic strategy for preventing disease progression into more irreversible forms characterized by the absence of adequate pharmacological treatment.

Dietary supplementation with virgin olive oil (VOO) at either low or high dose in rats caused a significant decrease in the serum triglycerides, total cholesterols, low density lipoprotein (LDL), and glucose, but increased high density lipoprotein (HDL) levels. This positive effect was attributed to its higher content of MUFAs (such as oleic acid), which exerted beneficial effects on the cardiovascular system of the male albino rats (Farahat et al., 2019). The MUFAs content of the olive oil exhibited an important role in modulating atherosclerosis, which ultimately affected the lipid profile and peroxidation in the hepatic mitochondria of rabbit (Massimo et al., 2009; Qian et al., 2016).

### Benefits of Olive Oil on Kidney

Natural compounds and medicinal herbs are playing a vital role in the control and prevention of many communicable and viral diseases (Dhama et al., 2018; Tiwari et al., 2018). It is well known that the VOO effectively inhibits the progression of nephrotoxicity induced by various chemical agents as dietary VOO and olive leaf extract have shown to control the nephrotoxicity in animal models (Abdel-Gayoum et al., 2015; Khalatbary et al., 2017). Cyclosporine may cause side effects like nephrotoxicity and the scientific community is searching for its replacement with some natural agents. It was found that the combined use of olive oil (1.25 ml/kg/d virgin olive oil) and naringenin, (100 mg/kg/d naringenin), respectively, reduced the cyclosporine-induced (25 mg/kg/d cyclosporine) nephrotoxicity by improving renal functionality and reducing the concentration of serum urea and creatinine in rats during a 45 days treatment period (Elshama et al., 2016). In another study, EVOO (2 ml/kg/day) reduced the signs of nephrotoxicity in rats exposed to ethopon (150 mg per kg daily) and improved the antioxidant and health status (Mokhtari et al., 2020). Moreover, administration of EVOO markedly reduced the tumor necrosis factor α (TNFα), interleukine-1, interleukine-6, uric acid, creatinine and urea levels in the serum and reduced the lethal effects caused by mercuric chloride (HgCl_2_) in the kidney (Necib et al., 2013).

According to Nekooeian et al. (2011), the control of nephrotoxicity and other diseases by olive oil feeding may be attributed to its phenolic compounds which behaved as potent lipid peroxidation inhibitors in addition to its ability to work as chelators which prevented toxicity by detoxifying metal ions. Various reports suggested that dietary VOO or its derived products have the therapeutic ability to address kidney related diseases in animals (Abdel-Gayoum et al., 2015; Khalatbary et al., 2017).

Human and experimental animals are contagiously suffering from renal toxicity due to the excessive use of a large number of pesticides and drugs (Ullah et al., 2018). Amikacin (a semisynthetic aminoglycoside antibiotic) is used to treat Gram-negative infections; however, it is known to cause nephrotoxicity (Abdel-Gayoum et al., 2015). In a study on rats, amikacin significantly increased the serum values of urea and creatinine, while co-administration of OLE and VOO significantly decrease their values, providing protection against amikacin-induced nephrotoxicity (Abdel-Gayoum et al., 2015).

### Anti-Neurodegenerative Effects

Neurological diseases like stroke, Parkinson’s and Alzheimer’s diseases, are serious concerns for human health. These ailments share frequent pathological appearances, such as the induction of inflammation, abnormal protein aggregation, apoptosis, oxidative stress, excitotoxicity and perturbed Ca^2+^ homeostasis. There is a large body of evidence supporting the favorable impacts of the Mediterranean diet in the prevention of neurodegeneration in humans (Angeloni et al., 2017). Mediterranean diets impart beneficial effects on human health owing to its rich polyphenolic contents of EVOO (Angelon et al., 2017). Notably, olive oil has been reported to have a positive influence on Parkinson’s Disease as the polyphenols present in olive oil can modify a different cellular mechanism involved in the onset and development of the disease (Sarrafchi et al., 2016; Maher, 2017).

The most critical and active component of olive oil is oleuropein, which has shown to reduce the cell damage, apoptosis and oxidative stress in PC12 cells induced by 6-OHDA in an *in vitro* model of Parkinson’s disease. Moreover, olive extract (400 and 600 μg/ml) or oleuropein (20 and 25 μg/ml) exhibited neuroprotective effects on PC12 cells subjected to 150 μM 6-OHDA. (Pasban-liabadi et al., 2013). Similarly, HTyr is another active component of olive oil which has shown to reduce the 5-S-cysteinyl-dopamine levels induced by monoamine oxidase inhibitors, which supported the clinical therapy of the Parkinson’s disease (Vauzour et al., 2010). Since most of the investigations have been conducted in cell cultures, *in vivo* experiments are required to prove the protective impacts of phenolic contents of olive oil that have been detected under *in vitro* studies. Furthermore, it would be essential to detect the protective impact of this oil in Parkinson’s disease.

Multiple Sclerosis (MS) is a complicated neurodegenerative ailment of the central nervous system that causes inflammation, axonal and oligodendrocyte injury, blood-brain barrier breakdown, demyelination and gliosis (Browne et al., 2014). Globally, the prevalence of MS has increased substantially from 2.1 million in 2008 to 2.3 million in 2013 (Browne et al., 2014) and it represents the first reason for disability in young individuals after traumatic brain injury (Sospedra, 2005).

Olive oil is suggested to be a powerful tool to cure and treat MS disorders in patients (Riccio et al., 2010). Similarly, Amyotrophic Lateral Sclerosis (ALS) is a progressive complex of neurological diseases refers to spinal cord muscle weakness and adversely affects the brain, causing paralysis and death due to constriction of respiratory muscles (Shneider, 2001). In this perspective, olive oil intake has been reported to delay the onset of ALS, while improving the motor performance and increase muscle fiber area. Moreover, supplementation of (20%, w/w) extra virgin olive oil folive oil upgraded the muscle status as confirmed by the augmented expression of myogenic regulatory factors (MRFs) (MyoG and MyoD) and reduced endoplasmic reticulum stress (Oliván et al., 2014).

De Paola et al. (2016) suggested that the phenolic extract derived from the commercial olive oil may modulate Toll-like receptor 4 signalling pathway involved in the pathogenic mechanisms of ALS. Furthermore, it is well endorsed that phenols of olive oil were able to provide neuroprotective impacts associated with modulation of inflammation. The positive impacts of olive oil or its phenolic components on the neurological problems through addressing different cellular pathways have been widely studied. Olive oil and its essential compounds such as oleuropein, ployphenol, HTyr, tyrosol and oleocanthal have been frequently investigated regarding their effects on the spinal cord and acute brain injuries. Additionally, tyrosol, oleuropein, and HTyr have been shown to decrease apoptosis, infarct volume and mitigate the outcome of these damages.

Finally, olive oil has various potent effects including antimicrobial, antioxidant, immunomodulatory, anticancer, anti-inflammatory, hepatoprotective, anti-neurodegenerative, neuroprotective, and other beneficial health effects. We summarized the biological effects and health benefits of olive oil in Table 1.

**TABLE 1:** Biological effects and health benefits of olive oil.

Activities	Results/Mechanisms	References
Antioxidant position, and antimicrobial activity	Phenolics of EVOO positively influence the inflammation, antioxidant position, and antimicrobial activity	Cicerale et al. (2012), Dick (2018)
	EVOO is much resistant to high temperatures and contains essential antioxidant compounds which scavenge free radicals, hence perform anti-inflammatory roles	
Antiobesity and Anti-hyperglycemic effect	The excessive use of conventional oil in food products increased body weight, and a plethora of obesity related diseases. Olive oil has been found beneficial in counteracting the obesity-related diseases	Abo-Omar (2000); that; Scoditti et al. (2019)
	Moreover, the dietary 5% olive oil increased serum HDL concentration, but decreased triglyceride level	
Immunomodulatory	Polyphenols of olive oil modify the immune system by increasing white blood cell proliferation, as well as by synthesis of cytokines and other factors, which contribute to immunological resistance	Psaltopoulou et al. (2011)
Anti-neurodegenerative effects	Phenolic extract derived from the commercial olive oil may modulate Toll-like receptor 4 signal pathway involved in the pathogenic mechanisms of Amyotrophic Lateral Sclerosis	De Paola et al. (2016)
Antimicrobial effect	Olive oil has a high quantity of phenols, which have strong antimicrobial and antioxidant compounds capable to reduce the growth and propagation of several bacteria. A remarkable antimicrobial response with the intake of olive extract has been attributed to its polyphenolic contents	Ritchason (2000), Cayan and Erener (2015)
Antioxidant and renoprotective	EVOO (2 ml/kg/day) reduced the signs of nephrotoxicity in rats exposed to ethopone (150 mg per kg daily) and improved antioxidant and health status. Various reports suggested that dietary virgin olive oil or the products derived from it have the capability of treating kidney related diseases in animals	Abdel-Gayoum et al. (2015), Khalatbary et al., (2017), Makhtari et al. (2019)
Antioxidant and Neuroprotection	Oleic acid in the olive oil played an important role in modulating atherosclerosis, which ultimately affected the lipid profile and peroxidation in the hepatic mitochondria of rabbit	Qian et al. (2016)
Growth enhancer and immunomodulatory effect	Higher antibody titers against Newcastle disease and improvement in growth and development were observed in the broilers given a diet enriched with olive oil relative to the control group	El-Bahra and Ahmed (2012), Tufarelli et al. (2016)
	EVOO significantly improved the BW gain, and feed efficiency, but reduced lipid peroxidation level of the chicks mainly through strengthening the antioxidant defense system	
Anti-cancer and antitumor	The dietary olive oil has been associated with a reduced number of cancerous lesions. Also, the fatty acids present in olive oil reduce the prostaglandins production obtained from the arachidonic acid that, in turn, plays an integral part in the tumor development and production	Nakbi et al. (2010), Grosso et al. (2013)
Anti-cancer effect	Diets enriched with EVOO have shown to limit the prevalence of several cancer types such as breast, prostate and digestive system cancer	Psaltopoulou et al. (2011)
Hepato-protective effect	EVOO contains oleic acid and polyphenols, which have been reported to exert protective impacts on the liver in several experimental models, particularly in animal studies and cellular cultures. These components of olive oil have been reported to prevent various hepatic disorders, like hepatic fibrinogenesis, hepatocyte ballooning, and liver steatosis, hence aid in prevention of hepatic tissue damage	Han et al. (2016), Pirozzi et al. (2016)

### Application of Olive Oil and Derivatives in Poultry Nutrition

**FIGURE 2:**
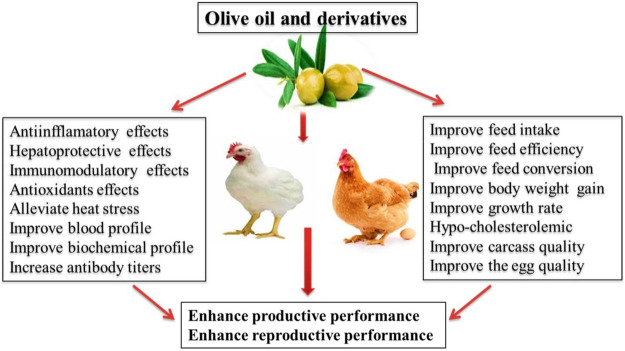
Application of olive oil and derivatives in poultry nutrition.

Health benefits and potential uses of olive oil and derivatives in poultry nutrition are illustrated in Figure 2. The phenolic components and carotenoids found in olive oil are naturally lipophilic and hydrophilic (Mahmoud et al., 2013). The phenolic components of olive oil such as oleuropein or HTyr induce antioxidant action in the gastrointestinal tract and its metabolites can effectively exhibit antioxidant properties (Omar, 2010). The protective impacts of oleuropein against H_2_O_2_-induced apoptosis was confirmed in human liver cells where the significant increase in expression of superoxide dismutase (SOD1), catalase, and glutathione peroxidase 1 were observed (Shi et al., 2017). In broilers, the supplementation of olive oil improved the growth rate (BW and body weight gain) and antibody titers against Newcastle disease virus as compared with the control group (El-Bahra and Ahmed, 2012).

Plant derived materials contain plenty of polyphenolic content, which positively affect the growth performance of the poultry (Abd El-Hack et al., 2020a,b; Abo Ghanima et al., 2020; Alagawany et al., 2020b; Ebrahim et al., 2020). No adverse effects on the carcass characteristics, inner body organs, growth performance and blood profile were observed in response to the feeding of olive cake (OC) in broilers (Zangeneh and Torki, 2011). Similarly, El Hachemi et al. (2007) reported that OC might successfully be included up to 15% without harmful effect on the feed intake and feed efficiency. On the other hand, significantly higher feed efficiency was obtained by the inclusion of 10% OC in broilers diet (Al-Shanti, 2003). Similarly, Al-Harthi (2017) reported a positive impact of including OC in poultry diet from 1 to 28 days trial period. Further, discussing the potential effects of OC, it was observed that its inclusion maintains a remarkably higher survival rate owing to its valuable nutrients such as essential fatty acids, essential amino acids, polyphenols and important elements which may be factors for this appreciable change.

In commercial poultry production systems, stress (induced by many factors such as, environmental, pathogenic and nutritional factors) negatively affects the growth and health of the birds. In contrast, *oxidative stress* ruins animal productivity by damaging the body proteins, lipids, DNA, and cellular structures (Lykkesfeldt and Svendsen, 2007).

The products derived from olive contain various vital nutrients like organic compounds, sugars, oils, fiber contents and polyphenols which may be recycled. The extract of olive leaves can reduce the dependency of poultry on grains and may reduce the competition for grains between human and animals (Tabera et al., 2004). Olive meal (OM) is yielded as a by-product during olive oil processing. The OM contains hefty amounts of lipids, including 73% oleic acid, 13% palmitic acid and 7% linoleic acid, which make it as an economical feed ingredient for animal (Ranalli et al., 2002). Preliminary findings revealed feeding of OM up to 9% exhibited no harmful effect on the growth performance while significantly improved the fiber intake in broilers (Zangeneh and Torki, 2011). Since OM is a waste of olive processing, its utilization as a feed ingredient in commercial poultry is an environment friendly technology particularly under resource limited set ups (Sateri et al., 2017).

Inclusion of 2% olive oil in feed facilitated the birds to gain higher BW with a better feed conversion ratio (FCR). Moreover, it significantly lowered the blood cholesterol, triglycerides and LDL, but elevated the level of HDL in chicken (Hadi and Al-Khalisy, 2018). Moreover, the addition of 400 ppm oleuropein promoted feed conversion efficiency of Japanese quails (Bahşi et al., 2016). Furthermore, feeding of olive oil resulted in an increased anti-inflammatory activity in chicken (Korver et al., 1998; Berbert et al., 2005). Similarly, broilers supplemented with olive extract led to an increased BW, as well as improvement of FCR (Erener et al., 2009). Similar incremental effects of feeding olive oil have been observed in the treated group when compared to control diet (Mujahid et al., 2009). In another study, the dietary (86 g/kg) supplementation of olive pulp did not show any adverse effects on production parameters of laying hens (Zarei et al., 2011). Moreover, Abo-Omar (2000) reported that the dietary olive oil (5%) increased serum HDL concentration, but decreased triglyceride level.

In another study, Ahmed et al. (2013) investigated the performance, immunity and biochemical profile of broilers by supplementing two sources of oil, i.e. olive and canola with four diets *viz*. control (no test diet), diet II (2% canola oil), diet III (2% olive oil), diet IV (1% olive oil + 1% canola oil). The significantly higher antibody response was observed with diet IV at 3, 7 and 10 days. Olive oil also alleviated the lethal effects of heat stress during hot climatic conditions by promoting the immunity of the birds. Furthermore, olive oil has shown to down-regulate the oxidative damages from heat stress and modified respiratory chain in the mitochondria of skeletal muscle. Moreover, liver enzyme activity was significantly higher in the birds fed with diet containing olive and canola oils. Additionally, the combination of olive and canola oils enhanced the weight gain and feed efficiency of the birds. In another study, the administration of olive oil in the rats intoxicated with 2-4 dichlorophenoxy acetic acid impacted the concentration of ALT and AST (Nakbi et al., 2010; Ahmad et al., 2013).

The dietary inclusion of processed olive pulp improved the FCR in broilers (Sayehban et al., 2016), mainly due to the presence of substances like flavanols, flavonoids, oleuropeosides and simple phenolic components (Herrero-Encinas et al., 2020). Zangeneh and Torki (2011) observed that olive leaf powder improved the color of egg yolk in layers. Furthermore, the phenolic components in olive leaf exhibited hypo-cholesterolemic activities by reducing the levels of hepatic and serum triglyceride, while modulating cholesterol metabolism (Sarica and Toptas, 2014).

It is well known that the oleuropein and HTyr derived from olive leaves inhibit LDL oxidation and decrease the secretion of an enzyme (3-hydroxy-3-methyglutaryl coenzyme A), responsible for the synthesis of cholesterol. This ability of oleuropein and HTyr may be attributed to the decreased yolk cholesterol concentration (Patrick and Uzick, 2001). The expressions of antioxidative enzymes while decreasing lipid peroxidation such as thiobarbituric acid-reactive substances content. Feeding of EVOO (2.5%) in male Hubbard broiler significantly improved the BW gain, and feed efficiency, but reduced lipid peroxidation levels mainly through strengthening the antioxidant defense system (Tufarelli et al., 2016).

## Conclusion

Studies reviewed in this article convincingly revealed that the use of olive oil and its bioactive molecules have shown a wide range of promising activities in various inflammatory and disease conditions. Olive oil may be a consistent approach to prevent and manage nutritional and health disorders. Besides, it may be used as an anti-inflammatory, anticancer, antimicrobials, hepatoprotective, renoprotective, and anti-neurodegenerative agent. However, further investigations are required to further explore the biological activities of olive oil and its derived compounds in poultry to improve bird health and produce enriched products.
